# Influence of Structure–Property Relationships of Polymeric Biomaterials for Engineering Multicellular Spheroids

**DOI:** 10.3390/bioengineering12080857

**Published:** 2025-08-09

**Authors:** Sheetal Chowdhury, Amol V. Janorkar

**Affiliations:** Department of Biomedical Materials Science, University of Mississippi Medical Center, 2500 N State Street, Jackson, MS 39216, USA; schowdhury@umc.edu

**Keywords:** multicellular spheroids, polymer, hydrogel, scaffold, ultra-low attachment surfaces

## Abstract

Two-dimensional cell culture systems lack the ability to replicate the complex, three-dimensional (3D) architecture and cellular microenvironments found in vivo. Multicellular spheroids (MCSs) present a promising alternative, with the ability to mimic native cell–cell and cell–matrix interactions and provide 3D architectures similar to in vivo conditions. These factors are critical for various biomedical applications, including cancer research, tissue engineering, and drug discovery and development. Polymeric materials such as hydrogels, solid scaffolds, and ultra-low attachment surfaces serve as versatile platforms for 3D cell culture, offering tailored biochemical and mechanical cues to support cellular organization. This review article focuses on the structure–property relationships of polymeric biomaterials that influence MCS formation, growth, and functionality. Specifically, we highlight their physicochemical properties and their influence on spheroid formation using key natural polymers, including collagen, hyaluronic acid, chitosan, and synthetic polymers like poly(lactic-co-glycolic acid) and poly(N-isopropylacrylamide) as examples. Despite recent advances, several challenges persist, including spheroid loss during media changes, limited viability or function in long-term cultures, and difficulties in scaling for high-throughput applications. Importantly, the development of MCS platforms also supports the 3R principle (Replacement, Reduction, and Refinement) by offering ethical and physiologically relevant alternatives to animal testing. This review emphasizes the need for innovative biomaterials and methodologies to overcome these limitations, ultimately advancing the utility of MCSs in biomedical research.

## 1. Introduction

**Figure 1:**
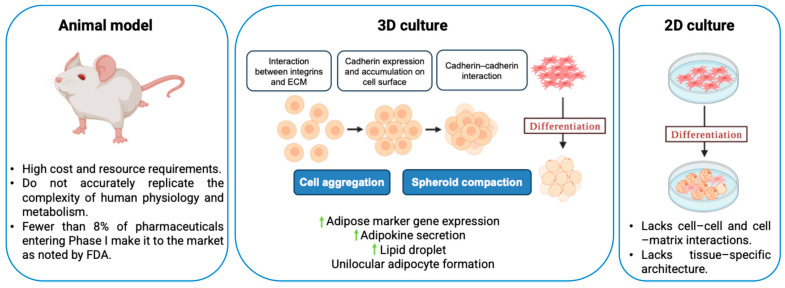
Models to recapitulate the underlying mechanisms of cellular behavior. Using adipocytes as an example, the figure illustrates three experimental models used to study cellular processes: in vivo animal models, providing a more complex, systemic context for examining cellular interactions in the living organism; 2D in vitro cell culture, representing a simplified cellular environment; and 3D in vitro cell culture, serving as an intermediate approach that bridges the gap between 2D culture and in vivo systems by mimicking the spatial and mechanical properties of tissues. These models collectively offer insights into the mechanisms of cellular behavior, providing a more comprehensive understanding of biological processes.

Two-dimensional (2D) cell culture has been extensively used to study cell biology and for drug development purposes. In 2D culture, one side of the cells is exposed to a rigid tissue culture plastic surface, and the other side is exposed to a liquid medium, thereby creating an environment different from the innate extracellular matrix (ECM) environment (Figure 1). This non-physiological environment requires cytoskeleton readjustment, leading to abnormal cell metabolism and protein expression. Because of the absence of cell–cell and cell–matrix interactions, as well as the loss of tissue-specific architecture, mechanical cues, and chemical cues, which are all crucial for the distinct functions of tissues in the human body, the 2D cell culture is unable to replicate the microenvironment and cell behavior present in vivo [1,2]. On the other hand, animal models have served to be more relevant compared with 2D cultures in terms of replicating complex biology, but they suffer from high cost and resource requirements [3]. Based on the 3R principle, Replacement, Reduction, and Refinement, ethical review boards encourage researchers to replace animal use with alternative methods, such as in vitro systems, where possible, reduce the number of animals used, and refine procedures to minimize the pain and distress caused to the animals. While originally introduced in the context of animal testing ethics in regenerative medicine and tissue engineering, the 3R principle also aligns with the broader goals of cost reduction and translational accuracy in drug discovery and oncology [4]. Additionally, animal models may not accurately replicate the complexities of human physiology and metabolism, which may be a factor in why fewer than 8% of pharmaceuticals entering Phase 1 clinical trials make it to the market, as noted in a Food and Drug Administration report [5]. The 3R ethical considerations and the inadequate replication of human physiology have accelerated the development of advanced in vitro models, to better replicate human physiology while minimizing the reliance on animal testing.

Cells in many in vivo tissues (e.g., adipocytes) occur in closely packed, three-dimensional (3D) clumps [6]. Recent advances in the in vitro 3D culture of cells, which tend to mimic complex cell-to-cell adhesion and cell–matrix interactions, use 3D spheroids with gradients of nutrients, gases, growth factors, and signaling molecules across the culture. Multicellular spheroid (MCS) formation involves three critical steps: (i) Dispersed cells are initially drawn closer to form loose clusters due to their long-chain ECM fibers with several arginine–glycine–aspartic acid (RGD) motifs that can bind strongly to the integrin on the cell membrane surface. (ii) Cadherin expression is increased when cells come into direct contact with one another as a result of early aggregation, leading to the accumulation of cadherin on the surface of the cell membrane. (iii) As a result of the homophilic cadherin–cadherin interaction, cells are compacted together to form solid aggregates known as MCSs [7].

The two strategies used for the preparation of 3D spheroid culture consist of matrix-free and matrix-dependent cell cultures, as discussed below. The matrix-independent techniques use liquid overlay, hanging drop, spinner flask, and magnetic levitation. The *liquid overlay technique,* also known as the ultra-low attachment (ULA) technique, utilizes a non-adhesive culture surface or the functionalization of the culture plate with biomaterials having low cell binding properties. This system prevents the attachment of the cells to the surface and forces the floating of the cells, thereby improving the cell-to-cell interaction, resulting in the formation of a multicellular aggregate [8]. The drawback of this technique is that the MCSs formed may be lost during media changes during long-term culture. *The hanging drop technique* is a simple technique where the cell suspension drops are placed on the underside of the lid of the culture plate, followed by an inversion of the lid. The gravitational force causes the cells to localize at the bottom of the drop, whereas the surface tension keeps the drop intact [8]. The drawback of this technique is that the MCSs formed need to be transferred prior to post processing. Additionally, the volume of media used is very low (typically < 50 μL); therefore, there is a chance of evaporation of the media during long-term culture. The *spinner flask technique* involves the continuous spinning of the media, allowing for cell–cell interaction by preventing the settling of the cells, thereby leading to MCS formation. It is important to control the spinning speed as lower speeds can cause the settling of the cells, whereas higher speeds can cause shear stress on the cells [9]. The drawback of this technique is that during continuous spinning, it is difficult to visualize the spheroid formation, an important factor in monitoring the morphology and size. *The magnetic levitation technique* involves loading the cells with magnetic nanoparticles and then directing the spheroid formation by the application of a magnetic field that causes cell–cell adhesion. While the incorporation of magnetic nanoparticles does not affect the cell behavior or inflammatory response within a 30–500 G magnetic field, a higher magnetic field of >800 G does influence cell behavior [10,11,12].

Matrix-dependent MCS formation techniques, also referred to as scaffold-dependent MCS formation techniques, use bio-mimicking matrices that induce cell–matrix interactions. There is a bidirectional interaction between the cells and the matrix, wherein the cells secrete matrix metalloproteinases (MMPs) that remodel the surrounding polymers and the polymers in turn exert mechanical forces on the cells, causing changes in the biochemical signals and thus changing the gene expression through mechanotransduction. The main goal of this technique is to mimic the properties of the ECM while maintaining precise control over experimental parameters such as cell numbers, porosity, and mechanical stiffness. The unique characteristic of the matrix-dependent technique is that the scaffold structure, morphology, and components can be adjusted to cater to the microenvironment required for MCS formation [13,14,15].

An important distinction among the materials used in spheroid culture platforms is their functional role; some act solely as inert scaffolds providing physical confinement, while others actively influence cellular behavior. For example, agarose and polyethylene glycol (PEG)-based microwells promote spheroid formation primarily by restricting cell migration without contributing to biochemical signaling. In contrast, biointeractive polymers such as collagen, gelatin, hyaluronic acid, and fibrin mimic ECM components and actively engage in cell adhesion, migration, and differentiation. This functional bifurcation, between spatially confining and bioactive materials, has significant implications for the choice of materials in designing 3D culture systems and interpreting their biological outcomes. Nevertheless, cells grown in a 3D microenvironment show realistic morphologies and express genes that are not well expressed in 2D culture conditions, underscoring the critical role of 3D culture systems in recapitulating in vivo-like cellular behavior. MCS models are now widely used across disciplines, not only in tissue engineering but also in cancer biology, toxicology, and pharmacology, for applications such as tumor microenvironment modeling, high-throughput drug screening, and evaluating therapeutic responses. Hao et al. recently reviewed the advances in three-dimensional multicellular platforms, reinforcing the importance of biomaterial selection in applications ranging from disease modeling to personalized therapeutics [16].

While existing review articles provide comprehensive insights into the various techniques for generating 3D spheroids and their wide-ranging applications [8,17,18,19,20,21], a critical gap persists in the detailed discussion of the physicochemical properties of the biomaterials used for spheroid generation. Polymeric biomaterials serve as a cornerstone in 3D cell culture by providing structural and biochemical cues that support cellular growth and organization. Various polymeric materials have been employed in 3D cell culture platforms to enhance culture efficiency and cellular functionality. These materials are utilized in diverse forms, including hydrogels, solid scaffolds, and ultra-low attachment plates, each offering unique advantages and limitations. In this review, we aim to explore the diverse range of polymers utilized in different 3D culture platforms. We will delve into their physicochemical properties and investigate how these attributes influence MCS formation, growth dynamics, long-term retention, and functionality. Additionally, we will highlight the structure–property relationships that govern the interactions between polymers and cells, providing a comprehensive understanding of their role in advancing 3D culture technologies for tissue engineering, drug development, and regenerative medicine applications.

## 2. Polymers Used in Hydrogels and Scaffolds for 3D Culture

Hydrogels and scaffolds play a crucial role in the development of 3D cell culture systems, offering a supportive environment that mimics native ECM conditions for cellular growth and differentiation. The ECM environment is essential for allowing the complex cell–cell and cell–matrix interactions required for spheroid formation. Unlike traditional 2D culture systems, which constrain cells to a flat surface, hydrogels provide a scaffold structure that encourages cells to aggregate into clusters. The hydrophilic nature of hydrogels minimizes adhesion to the substrate, allowing cells to migrate and interact more freely with one another. These interactions are the foundation for cellular aggregation, a critical step in the early stages of spheroid development [22].

**Figure 2:**
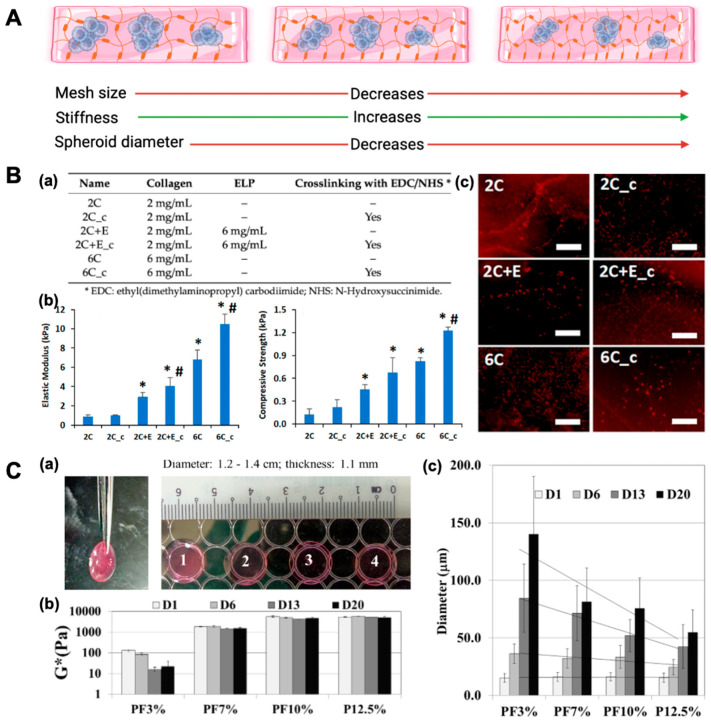
Effect of hydrogel/scaffold composition on mechanical properties, morphology, and adipogenic differentiation. (**A**) Schematic illustrating how increasing matrix density/crosslinking compacts the network (porosity ↓), elevates stiffness (↑), and limits spheroid size (↓). (**B**) Scaffold composition and impact on adipogenesis of human adipose-derived stem cells (hASCs). (**a**) Scaffold compositions detailing variations in collagen concentration, elastin-like polypeptide (ELP) addition, and EDC/NHS crosslinking. (**b**) Mechanical characterization of hydrated scaffolds showing an increased elastic modulus and compressive strength with a higher collagen content, ELP incorporation, and chemical crosslinking. Error bars represent 95% confidence intervals (* *p* ≤ 0.05 vs. 2C; # *p* ≤ 0.05 vs. non-crosslinked scaffold of same formulation). (**c**) Adipogenic differentiation of hASCs encapsulated in scaffolds visualized by Oil Red O staining for lipid accumulation on days 5 and 11. Scale bar: 200 μm. (**C**) Mechanically tunable polyethylene glycol-diacrylate (PEGDA) hydrogels support time-dependent Huh7.5 spheroid formation. (**a**) All the Huh7.5 cell-laden hydrogels exhibited viscoelastic properties. (**b**) Stiffness mimicking normal (soft) and cirrhotic (stiff) liver environments modulated Huh7.5 mechanotransduction and spheroid growth. Increased PEGDA concentration significantly elevated the complex modulus (one-way ANOVA, *p* < 0.001), with no significant difference between PF10% and P12.5% (*p* > 0.05). (**c**) Spheroid size increased over 20 days in a stiffness- and time-dependent manner. One-way ANOVA showed significant differences in spheroid diameters across conditions from day 6 onward (*p* < 0.05 to *p* < 0.001), with PF10% and P12.5% differing significantly at days 6, 13, and 20 (*p* < 0.01). Reprinted from open-access publications [7,29].

Bioactive hydrogels, such as those composed of collagen, further enhance this process by providing biochemical cues that guide cellular behavior. Collagen, a natural ECM protein, contains binding motifs that can interact with cell surface receptors like integrins, thereby initiating signaling cascades that regulate cytoskeletal organization, adhesion, and motility [23]. These signals are crucial for cells to cluster together, compact, and maintain cohesion. In addition to biochemical signaling, the mechanical properties of hydrogels play a significant role in determining cell behavior. The stiffness and elasticity of hydrogels can be customized to mimic the specific mechanical environment of different tissues, which influences how cells migrate, aggregate, and self-organize into clusters, as shown in Figure 2 [24].

Hydrogels also have a porous and permeable structure, which facilitates the diffusion of nutrients, oxygen, and signaling molecules while allowing the removal of waste products. This permeability ensures that cells remain viable and metabolically active throughout the culture period. Over time, the physical proximity and biochemical communication within clusters promote cellular rearrangement, proliferation, and differentiation, leading to the formation of cohesive spheroids that closely resemble in vivo tissues in terms of architecture and function [22].

Overall, hydrogels offer a versatile platform that can be modified with additional ECM components, growth factors, or bioactive molecules to further enhance spheroid formation. For example, the inclusion of laminin or fibronectin can mimic specific tissue environments, while growth factors such as vascular endothelial growth factor (VEGF) or transforming growth factor-β (TGF-β) can guide the differentiation of cells within spheroids [25,26]. Recent work by Di Caprio and Burdick has emphasized the role of engineered biomaterials in guiding spheroid morphology, assembly, and function, highlighting how dynamic hydrogel platforms and ECM-mimetic cues can be used to fabricate spheroids into organized 3D tissue constructs [27,28]. This level of customization makes hydrogels an indispensable tool in tissue engineering, cancer research, and regenerative medicine, where spheroids are used to study disease models, test drug efficacy, and develop engineered tissues. By integrating biochemical cues, mechanical properties, and a supportive 3D structure, hydrogels not only facilitate the formation of cellular clusters and spheroids but also ensure that these structures are functional and physiologically relevant, advancing their application in biomedical research. A detailed discussion of the various polymers utilized in hydrogel formulations is provided in the following section of this review.

### 2.1. Natural Polymeric Biomaterials for Multicellular Spheroid Engineering

Natural polymers have emerged as versatile biomaterials for engineering multicellular spheroids due to their intrinsic biocompatibility, bioactivity, and structural similarity to the native ECM. Derived from proteins or polysaccharides, these materials provide essential biochemical cues and degradability profiles that support cell adhesion, proliferation, and differentiation. Polymers such as collagen, gelatin, alginate, hyaluronic acid, and chitosan have been widely employed to promote spheroid formation and to simulate the 3D microenvironment required for physiologically relevant tissue models. Their ability to present native ligands, respond to enzymatic remodeling, and maintain hydrogel structures under mild processing conditions makes them ideal candidates for applications in regenerative medicine, cancer modeling, and drug screening. In this section, we discuss commonly used natural polymers, emphasizing their material properties, biofunctional relevance, and role in supporting spheroid morphology and function.

#### 2.1.1. Collagen

Collagen is an abundant structural protein in human tissue formed from a proline-rich polypeptide triple helix structure. It is widely used to mimic the ECM for adherent cells due to the availability of integrin binding sites such as the glycine–phenylalanine–hydroxyproline–glycine–glutamic acid–arginine (GFOGER) peptide [30]. The 3D porous structure of collagen hydrogels allows for the sufficient transport of oxygen, nutrients, and metabolic waste inside the structure, which allows for the long-term culture of MCSs [31,32]. The biodegradation property of collagen polymers due to enzymatic digestion can be utilized to harvest the MCSs from the scaffolds. The characteristics of the collagen hydrogel can be influenced by the source (species, tissue, and anatomical location) and processing parameters, including the collagen concentration, gelation temperature, and gelation pH. The collagen concentration, both in vivo and in vitro, influences the mechanical properties and, thereby, the cellular behavior. Collagen hydrogels fabricated at a concentration of less than 4 mg/mL do not mimic in vivo tissues due to their non-physiological strength and microstructure, which also results in their inability to support microfabrication as bulk gels. On the other hand, the hydrogels prepared at concentrations greater than 20 mg/mL form dense fibrous structures that prevent efficient cell migration and viability. Ramanujan et al. and Erikson et al. assessed the effect of collagen concentration (10–45 and 10–20 mg/mL, respectively) on both diffusivity and fiber structure, and they observed a decrease in diffusion rate, fiber length, and organization with increased collagen concentration [33,34]. A higher collagen concentration has been correlated with a higher fiber density and reduced pore size, but has no effect on fiber diameter. The gelation kinetics of collagen are temperature dependent, and collagen molecules self-assemble rapidly at higher temperatures, leading to an unordered structure and a lower number of bundled fibrils, which in turn influences the mechanical properties of the hydrogel. Increasing pH or temperature promotes electrostatic interactions and fiber nucleation, thereby accelerating gelation that produces fibers with reduced diameters and networks with small pore sizes. Similarly, decreasing the ionic strength of the hydrogel produces a decreased fiber diameter and pore size [35]. Altogether, these parameters are essential to optimize the collagen hydrogel environment, ensuring it supports spheroid formation, cellular viability, and functional organization in 3D culture systems.

Newman et al. created six different elastin-like polypeptide (ELP)–collagen scaffolds by varying the collagen concentration (2 and 6 mg/mL), ELP addition (6 mg/mL), or the crosslinking of the scaffolds. They observed that a higher collagen concentration, the addition of ELP, and the presence of crosslinking reduced the swelling ratio while increasing the elastic modulus and compressive strength of the scaffolds (Figure 2). These scaffold properties influenced cell morphology, with the hASCs seeded in softer, non-crosslinked scaffolds exhibiting a spread morphology. Softer scaffolds, such as 2 mg/mL of collagen (2C) (elastic modulus of 0.8 ± 0.2 kPa; compressive strength of 0.12 ± 0.08 kPa) and 2 mg/mL of collagen–6 mg/mL ELP (2C+E) (elastic modulus of 2.9 ± 0.5 kPa; compressive strength of 0.45 ± 0.07 kPa) exhibited moderate adipogenic differentiation, as indicated by Oil Red O staining by day 11, due to the formation of a spread morphology. In contrast, stiffer scaffolds, including the 2 mg/mL collagen crosslinked (2C_c), 2 mg/mL of collagen–6 mg/mL ELP crosslinked (2C+E c), 6 mg/mL collagen (elastic modulus of 6.8 ± 1.0 kPa; compressive strength of 0.82 ± 0.05 kPa), and 6 mg/mL collagen crosslinked (6C_c) (elastic modulus of 10.5 ± 1.1 kPa; compressive strength of 1.22 ± 0.05 kPa) promoted significantly enhanced adipogenic differentiation. These stiffer scaffolds displayed greater Oil Red O staining, highlighting the critical role of scaffold stiffness in driving adipogenesis. The increased mechanical stiffness supported a spheroidal cell morphology, which facilitated higher differentiation compared with the spread morphology observed in softer scaffolds. The crosslinked elastin–collagen scaffolds also restricted cell spreading, resulting in a spheroid morphology that promoted enhanced adipogenic differentiation, as evidenced by Oil Red O staining [29]. Baharvand et al. introduced human embryonic stem cell (hESC) spheroids into collagen scaffolds that were differentiated into hepatocytes after 5 days of culture. It was observed that the hepatocytes mimicked the in vivo functions and characteristics [36]. When luminal cells with myoepithelial cells and fibroblasts were co-cultured inside collagen type I matrix for 7 days, heterotypic MCSs developed [37]. Another novel technique involved collagen microparticles with a size range of 10 to 20 µm, which were used to create viable MCSs. In this method, the collagen microparticles were created by emulsifying membrane droplets in a non-equilibrium state in a micro-channel. In this study, heterogeneous composite spheroids composed of hepatocytes and collagen particles were prepared on agarose-coated microchambers. This approach recreated a similar in vivo microenvironment due to the incorporation of the ECM component, i.e., the collagen in the MCSs [38].

#### 2.1.2. Hyaluronic Acid

Hyaluronic acid (HA) is a non-sulfated glycosaminoglycan of the proteoglycan complex with a high molecular mass (between 1 and 10 MDa). HA is a linear anionic acidic polysaccharide composed of D-glucuronic acid and N-acetyl-D-glucosamine-disaccharide repeat units linked by alternating β-1,3-glycosidic and β-1,4-glycosidic bonds. The molecular weight of HA, a highly hydrated polyanionic macromolecule, ranges from 100,000 Da in serum to 8,000,000 Da in vitreous solution. HA has the capability to interact with specific cell surface receptors, thus activating the cascade of cell signaling pathways, for example, the interaction of HA with CD44 promotes cell adhesion and migration [39,40]. HA can be modified in a variety of ways to change the properties of the final materials, including those that increase hydrophobicity and biological activity. Three functional groups—the glucuronic acid carboxylic acid, the primary and secondary hydroxyl groups, and the N-acetyl group (after deamidation)—are the targets of chemical changes in HA, which have been thoroughly reviewed [41]. Most frequently, esterification, amination, and carbodiimide-mediated processes have been used to modify carboxylates; etherification, divinylsulfone crosslinking, esterification, and bis-epoxide crosslinking have been used to modify hydroxyls. However, HA has inadequate mechanical properties and degrades quickly via oxidative species and enzymatic processes, which prevents it from being used in various bio-applications. For instance, it is important to match the rate of tissue development to the rate of scaffold degradation for tissue engineering applications. HA chains can be physically or chemically crosslinked to create hydrogels to overcome these limitations. By altering its structure and creating a hydrogel, the native HA can have its physicochemical characteristics, stability, and half-life improved. Covalent crosslinking of HA reduces its water solubility, causing the network to swell upon water addition until an equilibrium is reached. At this point, osmotic swelling forces are counterbalanced by the elastic forces of internal atomic bonds. The strength and degradability of the HA derivative can be adjusted by modifying the degree of crosslinking and the type of crosslinker used, allowing these biomaterials to be customized for specific tissue applications. Essentially, there are two approaches to perform HA crosslinking: either by pre-modifying the HA chains with functional groups likely to undergo crosslinking or by directly adding a crosslinker and creating the 3D network [42].

HA is found in many tissues, such as skin and cartilage, where it promotes cellular survival, migration, angiogenesis, and differentiation by the transduction of intracellular signals [43]. Being non-adherent, HA does not support tumor cell attachment, thereby favoring spheroid assembly [42]. Due to their cell proliferation and spheroid forming capability, along with their excellent viscoelastic properties, HA hydrogels efficiently mimic the stiffness of native tissues. Owing to their biological relevance and their ability to mimic the native brain environment stiffness, which is between 200 and 1000 Pa, HA-based hydrogels have been explored to study the multicellular tumor spheroid culture of glioblastoma (GBM) cells [44]. Nakod et al. cultured the U87 cell line and patient-derived D456 GSCs (GBM stem cells) in HA-methacrylate or RGD-modified HA-methacrylate hydrogels that resulted in the formation of multicellular tumor spheroids. The stiffness of these 10 mM HA hydrogels was measured to be 1.64 ± 0.13 kPa, which is comparable to native brain tissue (~0.2–1 kPa), thereby providing the cells with an environment similar to in vivo. These spheroids showed an increased expression of the stem cell markers compared with the cells grown in monolayer and suspension cultures [45]. In another study, Pedron et al. showed that the incorporation of HA induced the formation of GBM multicellular tumor spheroids in a 3D environment, which was not observed in the case of poly(ethylene glycol)-tetraacrylate [46]. HA hydrogels have been used to study various other tumor types, such as breast, colon, prostate, and ovarian. Prostate cancer cells (LNCaP PCa) harvested after 7 days of culture in the HA hydrogels showed a significant upregulation of VEGF_165_ and IL-8 expression along with a low proliferation rate which was like LNCaP growth in vivo [20]. Feng et al. synthesized various concentrations of HA-based hydrogels and seeded them with different concentrations of hASCs. They observed that 6 × 10^6^ ASCs/mL in 3% HA gel achieved the highest spheroid density with appropriate spheroid sizes (20–100 μm). The stem cell marker expressions were upregulated in hASC spheroids cultured in HA hydrogel in comparison to a monolayer culture [47]. Hu et al. created a 3D co-culture model of hASCs and endothelial colony forming cells (ECFCs) on HA hydrogels. They discovered that after incubation on the HA substrate, the morphology of the hASCs was restored. The co-culture system showed an increased secretion of cytokines (hepatocyte growth factor) in comparison with single-cell 3D culture or monolayer culture [48]. Mineda et al. conducted a study wherein hASCs were seeded in non-crosslinked HA hydrogels of varying concentrations (0%, 2%, 3%, 4%, 5%, and 10%). In hydrogels containing 2–3% HA, some hASCs and hASC spheroids settled at the bottom, subsequently proliferating on the surface. Conversely, in 4–5% HA hydrogels, hASCs did not settle and completed spheroid formation within 48 h, with minimal size changes thereafter. No spheroid formation occurred in the 10% HA gel. On the other hand, on non-adhesive surfaces, hASCs formed spheroids that continued to grow for 7 days, resulting in spheroids of highly variable sizes. These findings highlight that HA hydrogels exert a mechanical compressive force that regulates spheroid growth, which is absent in ultra-low attachment plates [49].

#### 2.1.3. Chitosan

Chitosan is a linear polysaccharide made up of N-acetyl-D-glucosamine (acetylated unit) and β(1-4)-linked D-glucosamine (deacetylated unit) that is randomly arranged along the polymer chain. Chitosan is produced by exposing the chitin shells of crustaceans, such as shrimp, to an alkaline solution, such as sodium hydroxide. A porous chitosan scaffold with a high degree of deacetylation has been developed for the MCS culture of cancer cells, and it showed improved cell attachment and proliferation. The low solubility of chitosan in water hampers its application in MCS formation; therefore, copolymers of chitosan have been developed to improve the solubility. A hydrogel scaffold composed of chitosan–alginate copolymers has been developed to culture prostate cancer cell MCSs, where the chemical structure of the polymer was similar to glycosaminoglycans, a vital part of MCS ECM, and showed that the scaffolds were biocompatible, biodegradable, and less immunogenic. A 3D network is created when chitosan-based hydrogels interact with one another in different ways. The primary interactions that create the networks of chemical hydrogels are covalent bonds made up of complementary chemical groups. Chitosan and a crosslinker are usually combined to create chemical hydrogels resulting in networks [50]. Secondary interactions, such as electrostatic attraction, hydrogen bonding, hydrophobic contact, and crystallization, create physical hydrogels [50].

A fibrous scaffold of chitosan–alginate coated with collagen was used to culture MCF-7 cells, where the porous structure optimally replicated the in vivo environment. Spheroid formation started after day 2 of culture, where the spheroids of 100 μm diameter were formed after 6 days. An enhanced growth rate and drug resistance were displayed by the cells growing within the scaffold [51]. Chitosan with a poly(L-glutamic acid) scaffold was developed to culture adipose-derived stem cells (ASCs) into multicellular spheroids for cartilage regeneration. The scaffold did not allow protein attachment, thereby causing the MCS formation of the ASCs. The ASCs formed spheroids of 100 μm diameter in 2 days, showed improved chondrogenic differentiation, and decreased the deposition of collagen type I [52]. Huang et al. investigated the spheroid formation of mesenchymal stem cells on chitosan and chitosan–hyaluronan membranes. They observed that spheroids formed on chitosan and chitosan–HA membranes maintained greater expression of stem cell marker genes of MSCs in comparison to culturing cells on a polystyrene dish [53]. Lin et al. created a porous 3D scaffold using a mixed solution composed of chitosan and cartilage ECM lyophilized to generate a composite construct followed by crosslinking by genipin and used it for hASC culture. After 14 and 28 days of culture, hASCs in the chitosan/cartilage ECM composite 3D scaffolds formed cell spheroids with significant glycosaminoglycan synthesis. On day 14, the mRNA expressions of cartilage-specific genes COL2A1 and ACAN showed the chondrogenesis of hASCs seeded in 3D scaffolds. On day 28, histology and immunohistochemistry revealed the presence of cartilage-specific macromolecules, such as collagen type II and proteoglycan, deposited in a surface layer of the composite scaffold with tangential, transitional, and lacunae-like structures [54].

Polymer composition, crosslinking, stiffness, and porosity play critical roles in hydrogel formation, directly influencing spheroid size and function. Morello et al. developed a thermo-sensitive chitosan–pectin semi-interpenetrating polymer network hydrogel, where chitosan gelation was induced by a weak base, β-glycerophosphate at 0.04 M, and pectin was incorporated to enhance stability. Three hydrogel formulations with different polymer concentrations were tested: high (2.77% *w*/*v*), medium (1.66% *w*/*v*), and low (1% *w*/*v*). The study demonstrated that higher polymer concentrations resulted in stiffer hydrogels (higher Young’s modulus), leading to the formation of larger but morphologically irregular spheroids, whereas lower polymer concentrations produced softer hydrogels, facilitating the growth of smaller, more uniform spheroids. The hydrogels exhibited open, highly interconnected porous structures, with pore sizes ranging from 150 to 220 µm, directly influencing permeability and nutrient exchange. The optimized medium-stiffness hydrogel supported colorectal cancer spheroids (HCT-116) for up to 44 days, with spheroid size and density being directly modulated by hydrogel stiffness and permeability [55]. Similarly, Chang et al. developed a chitosan–PEG–genipin (CSPG) hydrogel for glioblastoma spheroids, where chitosan was modified with PEG and crosslinked with genipin to enhance mechanical strength. This crosslinking stabilized the gel, increasing stiffness from 10 to nearly 1000 Pa over 24 h, closely matching the physiological stiffness of glioblastoma tissue (100–5000 Pa). Glioblastoma cell lines (U87-RFP and U118-RFP) cultured in CSPG hydrogels self-assembled into dense, compact spheroids with diameters reaching approximately 200 µm within 3–5 days, which was significantly faster than spheroid formation in Matrigel. The spheroids exhibited increased chemoresistance when treated with temozolomide and carmustine compared with 2D cultures, as evidenced by higher lethal dose (LD_50_) values for both drugs [56].

#### 2.1.4. Matrigel

Matrigel is a commercial product derived from the basement membrane of Engelbreth–Holm–Swarm mouse tumors and has been widely used to study cancer biology. It is composed of several ECM components, including laminin, collagen IV, entactin, and heparan sulfate proteoglycan, as well as several growth factors such as fibroblast growth factor, epidermal growth factor, insulin-like growth factor-1, transforming growth factor beta, platelet-derived growth factor, and nerve growth factor [57]. These components collectively recreate a rich ECM-like environment that supports cell adhesion, migration, proliferation, and differentiation. Matrigel has been widely used to study MCS formation for PC-3M, PrCa, and NCI-H600 cells [58]. In one study, the HepG2 MCSs cultured on Matrigel showed an improved cell proliferation capability with larger MCSs compared with MCSs formed on collagen and gelatin hydrogels [59]. Despite its widespread utility, Matrigel presents several limitations. Its tumor-derived origin introduces lot-to-lot variability and potential immunogenicity, making it less suitable for translational and clinical applications. Furthermore, its undefined composition poses challenges for reproducibility and mechanistic studies where precise control over matrix components is required. While it remains a gold standard for mimicking native basement membrane environments, researchers have increasingly turned to synthetic or recombinant ECM substitutes to improve standardization and scalability. In conclusion, Matrigel plays a pivotal role in MCS research by providing a complex, growth factor-rich matrix that supports 3D tissue architecture and cellular function. However, its biological variability and limited clinical translatability necessitate the continued development of well defined and tunable ECM-mimetic biomaterials for more consistent and application-specific use.

### 2.2. Synthetic Polymeric Biomaterials for Multicellular Spheroid Engineering

Synthetic polymers offer tunable mechanical, chemical, and structural properties that make them attractive for engineering multicellular spheroids. Unlike natural polymers, synthetic materials allow for precise control over parameters such as stiffness, degradation rate, and functionalization, enabling the customization of 3D microenvironments to match specific biological applications. These polymers are often inert, non-immunogenic, and reproducible across batches, making them well suited for high-throughput screening and clinical translation. Moreover, advances in polymer chemistry have enabled the incorporation of bioactive motifs (e.g., RGD peptides) into otherwise biologically inert scaffolds, thereby enhancing cell–material interactions while retaining structural integrity. In this section, we explore the key synthetic polymers used in spheroid culture systems, highlighting their design flexibility, scalability, and compatibility with emerging fabrication techniques such as micropatterning and bioprinting.

#### 2.2.1. Poly(ethylene Glycol)

Poly(ethylene glycol) (PEG), because of its non-toxicity and non-immunogenicity, is one of the most common synthetic polymers used for 3D cell culture. PEG can be crosslinked using a variety of methods, including photopolymerization and emulsion polymerization. PEG-based hydrogels with varied mechanical characteristics were utilized to cultivate hepatocellular carcinoma cell lines (Huh7.5) for MCS development to investigate the impact of microenvironmental stiffness on cell aggregations. The stiffness of Huh7.5 cell-laden PEG-based hydrogels, determined by rheological measurements, was controlled by varying PEG-diacrylate (PEGDA) concentrations (3–12.5%). Higher PEGDA concentrations resulted in greater complex modulus values (*p* < 0.001). PEGDA-fibrinogen (PF)3% (3% PEGDA + 1% PF) had the lowest stiffness at 0.13 ± 0.01 kPa, followed by PF7% at 1.87 ± 0.07 kPa, PF10% at 5.72 ± 0.49 kPa, and P12.5% (12.5% PEGDA) at 5.59 ± 0.45 kPa. Spheroid size increased across all hydrogels, with PF3% supporting the largest growth (15.2 ± 3.7 μm at day 1 to 140.1 ± 50.2 μm at day 20), followed by PF7% (16.1 ± 3.8 μm to 81.4 ± 29.4 μm), PF10% (16.3 ± 3.5 μm to 75.8 ± 26.5 μm), and P12.5% (15.5 ± 4.2 μm to 55.0 ± 19.35 μm). Softer hydrogels (PF3% and PF7%) supported larger spheroid growth, while stiffer hydrogels (PF10% and P12.5%) exhibited smaller spheroids (*p* < 0.05). Larger spheroids were generated for the hydrogel network with higher compliance or lower stiffness. Additionally, it was discovered that compliant hydrogels improved spheroids’ cell proliferation, albumin secretion, and CY450 expression. This may be because a more compliant matrix allowed for better oxygen and nutrient diffusion [7]. For the growth and synthesis of submandibular gland MCSs, two distinct polymerization techniques—chain addition for methacrylate-based PEG and step-growth for thiolene polymerization—were applied. Due to reduced membrane peroxidation and the generation of intracellular reactive oxygen species, step-growth thiolene polymerization performed better in terms of cell viability, proliferation, and aggregation [60].

#### 2.2.2. Poly(lactic-co-glycolic Acid)

Poly(lactic-co-glycolic acid) (PLGA) is generally synthesized by the ring-opening copolymerization of two distinct monomers, glycolic acid and lactic acid cyclic dimers. Additional polymer properties can be added by the synthesis of random or block copolymers. Tin(II) 2-ethylhexanoate, tin(II) alkoxides, or aluminum isopropoxide are often employed as catalysts in the production of this polymer. In PLGA, subsequent monomeric units of glycolic or lactic acid are connected by ester linkages during polymerization, producing a linear, aliphatic polyester as a byproduct. This polymer has received a lot of attention because of its biodegradability in 3D cell culture. This synthetic polymer degrades into natural metabolites (glycolic acid and lactic acid), ensuring biocompatibility and making it highly suitable for applications in 3D cell culture and other biomedical fields. PLGA has been used in various forms, including foam, fibers, and sponges [61]. To culture hepatocyte spheroids, a porous PLGA microsphere scaffold was created [62]. This strategy accelerated the creation of MCSs as the porous scaffold structure accelerated cell attachment and waste transfer. The PLGA scaffold performed well in recreating the environment needed for tumor engineering when MCF-7 and U87 cell lines were grown in it to create MCSs [63]. The microenvironmental traits of the tumor model created from the PLGA scaffold are similar to that of in vivo tumors. For cells cultivated in the PLGA scaffold, it was discovered that the expression of interleukin-8 (IL-8), an angiogenetic factor, was elevated [64]. The angiogenic characteristic was similar to that of in vivo tumors, and cells in this model were less sensitive to chemotherapy, demonstrating the tumors produced had improved malignant potential. To mimic a bone-like environment for the development of breast cancer spheroids, PLGA was combined with hydroxyapatite. Due to hydroxyapatite’s promotion of breast carcinoma cells’ neoplastic and metastatic growth as well as its stimulation of IL-8 release, this scaffold aided in the proliferation and aggregation of breast cancer cells [65].

#### 2.2.3. Poly(N-isopropylacrylamide)

Poly(N-isopropylacrylamide) (PNIPAM) can easily be functionalized and manufactured using free radical polymerization, making it valuable in a range of applications. Though PNIPAM dissolves in water, these solutions go through a reversible lower critical solution temperature phase transition from a soluble hydrated state to an insoluble dehydrated form when heated over their cloud point temperature. This phase transition is generally thought to occur at 32 °C (90 °F) and can be altered by 5 to 10 °C (or even more) depending on the polymer concentration, molar mass of polymer chains, polymer dispersity, and terminal moieties. Due to its thermal reversibility, which enables the harvesting of MCSs without the use of hazardous or powerful chemicals, PNIPAM hydrogel is another well liked polymer network. The PNIPAM containing copolymers, which were created through copolymerization with different monomers, can support various cell types, encourage cell proliferation and aggregation, and preserve tissue functionality. A microgel with a diameter of around 300 nm was shown to have improved cell proliferation and MCS production when the PNIPAM network was employed for HepG2 MCS culture [66]. For growing HepG2 MCSs, acrylic acid (AA) was used to modify the PNIPAM polymer. HepG2 cells proliferated most effectively in the hydrogel containing 1% AA in the copolymer, and PNIPAM-AA showed minimal shrinkage to allow long-term culture and to maintain the scaffold structure. The addition of 1% AA significantly improved cell viability and growth compared with the PNIPAM hydrogel that lacked AA. In the PNIPAM hydrogel that lacked AA, the number of viable cells did not increase over time due to high shrinkage, which reduced pore size and limited oxygen and nutrient diffusion, while also mechanically constraining the cells. In contrast, the PNIPAM-AA hydrogel exhibited a reduced degree of shrinkage, allowing for better nutrient and oxygen transport, resulting in a rapid increase in viable cells during the first 9 days. Although cell proliferation plateaued thereafter due to limitations in passive diffusion at high cell densities, the overall viability remained constant and high [67,68]. To culture HepG2 MCSs, PNIPAM-AA microgels were further galactosylated. The galactose ligands assisted HepG2 MCSs in carrying out liver-specific tasks with increased albumin secretion and urea synthesis compared with a hydrogel without galactose ligands [69]. Additionally, PEG was added to the PNIPAM hydrogel to improve cell adherence. To create spheroids, human pluripotent stem cells (hPSCs) were grown in the PNIPAM-PEG hydrogels. Spheroids made from hPSCs had high proliferative rates, kept their pluripotency under less-than-ideal culture conditions, and had high survival rates [70]. HepG2 cells were pre-aggregated before being cultivated in the hydrogel using this technique, which was also employed to culture HepG2 spheroids [71]. Sodium periodate (NaIO_4_) was used to give HepG2 cells surface aldehyde functions, while chitosan, an intercellular linker modified by acrylic acid, was used to aggregate the cells, which resulted in spheroid formation in 1 day. Kim et al. utilized a PEG hydrogel microwell-patterned PNIPAM hydrogel substrate to form uniform hASC spheroids between 100 and 150 μm in diameter on 200 and 300 μm well-patterned substrates. The resulting hASC spheroids were evaluated for cell–cell interactions and it was observed that fibronectin and laminin expression were higher in hASC spheroids than those of hASCs cultured on the tissue culture plate. The hASC spheroids were detached and the retrieved spheroids showed a cell viability over 97.5% [72]. Table 1 summarizes the selected studies.

## 3. Polymers Used in Low Attachment and Concave Platforms for 3D Culture

**Figure 3:**
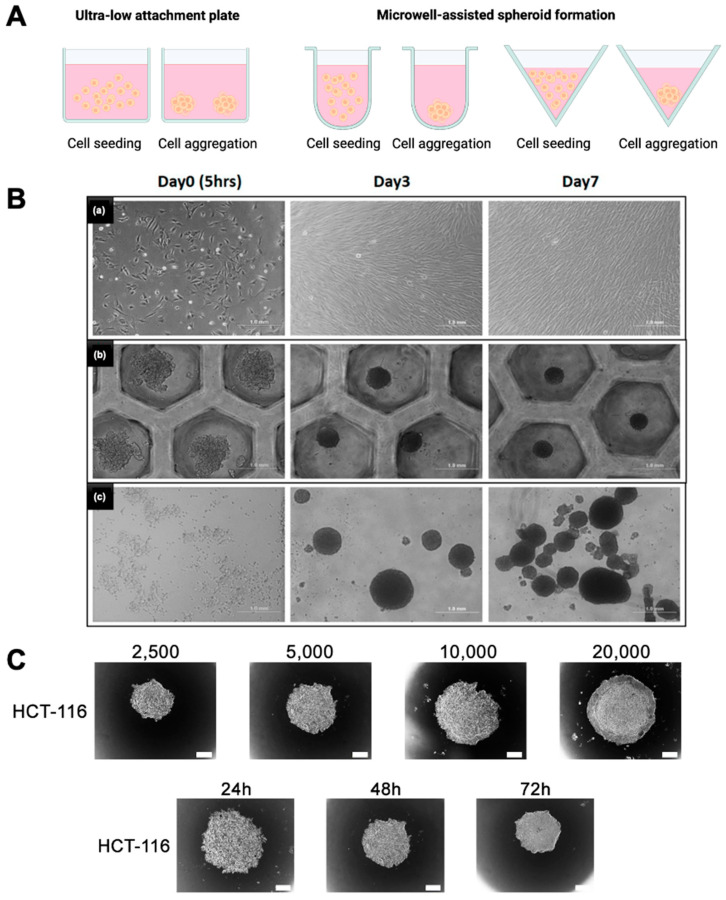
Comparison of spheroid culture methods. (**A**) Schematic representation of spheroid culture techniques, including ultra-low attachment (ULA) plate and microwell-based aggregation formats. (**B**) Brightfield images showing cell morphology and spheroid formation at day 0 (5 h), day 3, and day 7 for a (**a**) 2D monolayer culture, (**b**) microwell-based spheroid culture, and (**c**) spontaneous aggregation in ULA plates. Scale bar = 1 mm. (**C**) Effect of initial seeding density (2500–20,000 cells/well) and culture duration (24–72 h) on spheroid formation in HCT-116 cells using ULA plates. Spheroid diameter increases with both cell density and culture time. Scale bar = 100 μm. Reprinted from open-access publications [76,77].

ULA surfaces have become a cornerstone technology for the generation and maintenance of MCSs. Unlike traditional 2D cell culture systems, where cells spread and adhere to flat surfaces, ULA surfaces are engineered to minimize cell–substrate adhesion. By disrupting adhesion pathways, ULA surfaces promote the aggregation of cells, enabling them to form tightly packed, 3D structures through enhanced cell–cell interactions as shown in Figure 3. There are several commercially available ULA plates available in the market, such as Corning^®^ Clear Flat Bottom Ultra-Low Attachment Microplate, Revvity PhenoPlate optically clear flat-bottom ultra-low attachment coated plate, etc. The spheroids cultured on ULA closely mimic the architecture, microenvironment, and functional behavior of in vivo tissues, providing a more physiologically relevant model for studying cellular processes. A detailed discussion of the various polymers utilized in ultra-low/low adhesion or concave multi-well configurations is provided in the following section of this review.

Microwell-based techniques are among the most widely adopted methods for generating multicellular spheroids due to their simplicity, high-throughput compatibility, and reproducibility. These platforms utilize non-adhesive materials such as agarose, polydimethylsiloxane (PDMS), and PEG-based hydrogels to create concave or cylindrical microwells that spatially confine cells into discrete, uniform niches. Within these confined spaces, cells can self-assemble and form spheroids with defined diameters, thereby improving the consistency of biological assays. Importantly, the size of the microwells can be engineered to control spheroid dimensions, which is critical for modeling oxygen/nutrient gradients and drug diffusion kinetics in vitro. A variety of commercial microwell systems are now available and have been widely used in both academic and pharmaceutical research settings. For instance, platforms like AggreWell™, Elplasia^®^, and Sphericalplate 5D provide scalable and user-friendly formats for generating hundreds to thousands of uniform spheroids per well. These systems offer tunable well sizes, compatibility with automated liquid handling, and are particularly valuable for applications in cancer research, stem cell differentiation, and drug screening. Despite their advantages, microwell-based systems may limit long-term culture due to restricted diffusion in static conditions, which has led to innovations combining microwells with perfusion bioreactors or dynamic culture systems to overcome nutrient depletion and waste accumulation [73,74,75].

### 3.1. Agarose

Agarose, a linear polymer with a molecular weight of around 120,000 Da, is composed of 3,6-anhydro-L-galactopyranose and D-galactose that are alternately linked by α-(1→3) and β(1→4) glycosidic linkages. The 3,6-anhydro-L-galactopyranose is an L-galactose with an anhydrous bridge between the 3 and 6 positions with minor amounts of pyruvate, sulfate, and certain D- and L-galactose units that can be methylated [78]. Typically, the agarose chain includes around 800 galactose units, and these chains organize into helical fibers that aggregate into a supercoiled structure with a radius of 20–30 nm. These fibers are semi-rigid and can vary in length depending on the agarose solution concentration. When solidified, agarose forms a 3D network of channels with diameters ranging from 50 nm to over 1200 nm, with higher concentrations yielding smaller average pore diameters. Hydrogen bonds hold the 3D structure together, which can be disrupted upon heating, causing the disruption of the gel [79]. Fennema et al. (2018) utilized agarose micromolds with a diameter of 400 µm to generate ASC spheroids [80]. Guo et al. developed agarose micromolds using low-glucose Dulbecco’s Modified Eagle’s Medium (LG-DMEM) as a medium to produce 3D spheroids, which were later transferred to plastic Petri dishes. This innovative approach aimed to enhance post thaw cell viability and improve the neuronal differentiation potential of 3D ASC spheroids [81,82]. Similarly, Coyle et al. investigated different spheroid sizes (115, 135, 175, and 215 μm radius) and demonstrated, using in vitro culture and mathematical modeling, that glucose availability in the absence of oxygen improved spheroid viability with increasing spheroid size via anaerobic glycolysis [83]. De Moor et al. created an agarose microwell containing micropores, and cells were added to the well such that roughly 350 cells were present per pore. In this study the researchers investigated how ASCs, Human Umbilical Vein Endothelial Cells (HUVECs), and human foreskin fibroblasts (HFF) reacted to aggregation throughout a 10-day period. This study demonstrated the high-throughput fabrication of self-organized, vascularized, bioprinting-compatible spheroids, with ASC-containing spheroids, especially HUVEC/HFF/ASC, showing significantly higher angiogenic capacity than HUVEC/HFF spheroids [84].

### 3.2. Polydimethylsiloxane

Polydimethylsiloxane (PDMS) is an elastomeric polymer that has been widely used for biomedical applications due to its excellent biocompatibility, resistance to biodegradation, chemical stability, gas permeability, good mechanical properties, excellent optical transparency, and simple fabrication by replica molding. Since PDMS is non-biodegradable, it is suitable for in vitro applications where material degradation is not required. Its physical stability and surface tunability make it especially useful for generating reproducible spheroids in concave microwell systems. PDMS, due to its molecular structure, has a low surface energy and is hydrophobic. Hydrophobic surfaces show higher levels of protein adsorption than hydrophilic surfaces, thereby promoting increased cellular adhesion. Therefore, to support spheroid formation, an overnight treatment of PDMS surfaces with bovine serum albumin (BSA) was performed. This procedure leads to moderate surface wettability and the desired surface morphology, resulting in optimal surfaces with anti-fouling qualities that improve homogenous spheroid generation. Cho et al. used a similar approach to create spheroids in methylcellulose-coated supports in 96-well plates, which were then covered with BSA to prevent cell attachment [85]. No et al. coated the plates with 3% BSA, resulting in spheroids of a different diameter (500 μm), using PDMS-based concave micromolds developed using thin PDMS membranes and the soft lithography process. No et al. also discovered that hepatocyte spheroids co-cultured with hASC spheroids had increased liver-specific function [86].

### 3.3. Pluronic F127

Pluronic F127 (PF127) is a non-ionic triblock copolymer consisting of poly(ethylene oxide)-poly(propylene oxide)-poly(ethylene oxide) (PEO-PPO-PEO), which shows an amphiphilic character in an aqueous environment. The lower critical solution temperature of PF127 can be varied from 25 to 37 °C by changing the concentration of PF127 in the formulation. PEO is known to be hydrophilic, electroneutral, with a large excluded volume, and a unique ability to organize surrounding water molecules in an aqueous medium. PF127 utilizes these properties of PEO, where the large excluded volume can lead to a larger surface coverage at a lower concentration. The hydrophilic nature of PEO contributes to the ability of the PF127 to not allow the cell attachment [87,88,89]. Rumiski et al. used a matrix-free, low-adhesion strategy to manufacture ASC spheroids from plates treated with Pluronic F127 aqueous solution and compared their osteogenic potential to ASCs cultured in 3D porous polystyrene scaffold. ASC proliferation increased in polystyrene scaffolds, similar to the control 2D condition, whereas osteogenic marker expression was found to increase in scaffold-free ASC spheroids formed atop the Pluronic F127 coated surfaces [90]. Table 2 summarizes the selected studies.

## 4. Novel 3D Culture Techniques Using Polymer Coatings

Chen et al. engineered a chemically defined and mechanically robust surface modification platform to promote spheroid formation and the growth of hASCs [91]. Utilizing chemical vapor deposition (CVD), a copolymer of poly-para-xylylene was synthesized to present a N-hydroxysuccinimide (NHS) ester and maleimide functional groups in a defined 10:1 molar ratio. This configuration enabled the orthogonal and covalent immobilization of chitosan and fibroblast growth factor-2 (FGF-2) under aqueous, catalyst-free conditions, thereby generating a bioactive surface with tunable biochemical functionality. Chitosan immobilization supported cell–cell aggregation and spheroid assembly by limiting cell–substrate adhesion, potentially via the CD44 and integrin-mediated signaling pathways. On the other hand, surface-tethered FGF-2 enhanced cell proliferation within spheroids. Compared with the chitosan-only and unmodified tissue culture polystyrene (TCPS) controls, the dual-functionalized surface significantly increased spheroid size (~210 µm by day 10) and cell number, without compromising viability, as demonstrated by MTT assays and live/dead staining. Thorough physicochemical characterizations confirmed the uniformity, chemical composition, and functional group retention of the coating. The copolymer demonstrated excellent thermal stability up to 50 °C, strong adhesive strength (verified by scratch and tape-peel tests), and negligible leaching over 30 days, highlighting its suitability for long-term biological applications. Furthermore, spheroids formed on the modified surface exhibited a significantly elevated expression of pluripotency-associated transcription factors (Oct4, Sox2, and Nanog), as confirmed by qPCR and immunofluorescence analysis. These findings underscored the role of well defined polymeric interfaces in directing stem cell fate through the spatially controlled presentation of biochemical cues. The study provides a durable and modular biomaterial platform for spheroid culture, with implications for 3D in vitro modeling, stem cell expansion, and regenerative medicine [91].

Choi et al. introduced a vapor-phase polymer thin film system using poly(2,4,6,8-tetravinyl-2,4,6,8-tetramethyl cyclotetrasiloxane) (pV4D4), synthesized via initiated chemical vapor deposition (iCVD), to induce the spontaneous spheroid formation of various human cancer cell lines under adherent culture conditions [92]. Among several tested polymer coatings, only pV4D4 supported robust spheroid formation, highlighting its unique surface characteristics in guiding cell behavior. The iCVD-derived pV4D4 films exhibited a smooth topography, high conformality, and hydrophobic surface (water contact angle ~90°), while preserving thermal and mechanical stability. XPS and FTIR confirmed the successful polymerization of the V4D4 monomer and consistent surface composition. The non-fouling and moderately adhesive nature of pV4D4 was pivotal in facilitating cell–cell rather than cell–substrate interactions. The film’s surface properties facilitated weak but sufficient cell–substrate interactions, which supported initial attachment and viability while favoring cell–cell aggregation over cell spreading. This microenvironment promoted the rapid aggregation of epithelial cancer cells (e.g., SKOV3, MCF-7, Hep3B) into compact spheroids within 24 h, without requiring low-adhesion surfaces or external agitation. This study underscored the power of vapor-deposited polymer interfaces to modulate cell phenotype and organization through precisely engineered surface properties. Such platforms offer a chemically defined, scalable alternative for generating cancer stem cell (CSC)-enriched spheroids in cancer biology and drug screening applications [92]. Yu et al. utilized a vapor-deposited p(V4D4) thin film, previously developed by Choi et al., to induce spheroid formation in human mesenchymal stem cells (hMSCs) and enhance their therapeutic potential [93]. The surface was fabricated via initiated chemical vapor deposition (iCVD), producing a uniform, hydrophobic, and moderately adhesive coating (~100 nm thick). These surface characteristics supported initial cell attachment while restricting spreading, thereby promoting the rapid self-assembly of hMSCs into 3D spheroids under adherent culture conditions. Spheroids formed on poly(V4D4) exhibited a significantly elevated expression of pluripotency markers (Oct4, Nanog, and Sox2) and improved multilineage differentiation capacity (osteogenic, adipogenic, and chondrogenic) compared with 2D cultures. Increased E-cadherin expression was observed, suggesting strengthened cell–cell adhesion within the spheroids. Transcriptomic analysis further revealed the enrichment of genes related to stemness, cell adhesion, and differentiation. Functionally, hMSC spheroids formed on p(V4D4) showed enhanced bone regeneration in a murine calvarial defect model, confirming in vivo relevance. This study demonstrates that surface-driven 3D culture using chemically defined polymer coatings like p(V4D4) can potentiate stem cell functions without the need for biological coatings or suspension culture. It reinforces the importance of surface chemistry and controlled adhesion in directing spheroid formation and stem cell fate [93].

Elastin-like polypeptides (ELPs) are a family of polypeptides, with a molecular structure similar to that of mammalian elastin, which usually consists of the peptide sequence of VPGXG, where V = valine, P = proline, G = glycine, and X = any amino acid except proline. ELPs exhibit thermally induced phase transition behavior, characterized by reversible intramolecular contraction and intermolecular coacervation at the inverse transition temperature. To create a coating that would support the culture of hepatocytes while maintaining their differentiated state, Janorkar et al. synthesized ELPs using genetically modified *Escherichia coli*, modified to express ELPs with the primary sequence of H_2_N-MVSACRGPG-[VPGVG]_40_-WP-COOH, where A = alanine, C = cysteine, G = glycine, M = methionine, P = proline, R = arginine, W = tryptophan, and V = valine. The synthesized ELP was later modified with a polyelectrolyte, polyethyleneimine (PEI; Mw ~800 Da), by utilizing carbodiimide chemistry. The PEI used in this study had a branched structure, with a primary/secondary/tertiary amine ratio of 1:4:1, and each PEI molecule had approximately five primary amine groups available for reaction with the ELP. The positive charges on the PEI molecule induced spheroid formation, whereas the ELP provided a stable anchor for spheroid attachment to the surface. A total of 200 μL of 5 mg/mL of the ELP-PEI was coated on the 24-well TCPS plate, and 2 × 10^5^ rat hepatocytes were plated on the coating. It was observed that after 72 h, the cells aggregated to form spheroids, and the hepatocyte spheroids cultured on ELP-PEI exhibited significantly higher rates of albumin and urea production in comparison to those cultured on TCPS [94]. In another study, Turner et al. cultured 3T3-L1 mouse preadipocytes on 24-well TCPS plates coated with 200 μL of 5 mol% of 5 mg/mL of ELP-PEI. They observed the efficacy of the coating in creating a 3D adipocyte spheroid culture system and evaluated markers indicative of adipogenesis. It was observed that the cell response to exogenous oleic acid (OA) and linoleic acid (LA) treatments in terms of fat consumption in a 3D adipogenic culture system was comparable to that in a 2D monolayer. However, a sustained overall increase in intracellular triglyceride storage and fatty acid uptake indicated a differential advantage provided by the 3D spheroid culture system [95]. Turner et al. studied the adipogenic differentiation of hASCs grown as spheroids atop an ELP-PEI coating. It was observed that spheroid size grew during the maintenance phase; however, spheroids treated with LA or OA were smaller in size compared with those cultivated in the control maintenance medium. CD36 expression, which represents a cell’s ability to consume extracellular fatty acids, was consistently observed to be higher in 3D hASC spheroids than in 2D monolayers. The expression of the key adipogenic gene, PPAR-γ, was also elevated after the fatty acid treatment in 3D culture. Therefore, the ELP-PEI coatings proved to be a novel technique where the ELP allows cell attachment and PEI induces spheroid formation. This coating can be used to tether spheroids to the coatings, thereby allowing the long-term culture of the spheroids [96].

## 5. Summary of the State of the Art

**Figure 4:**
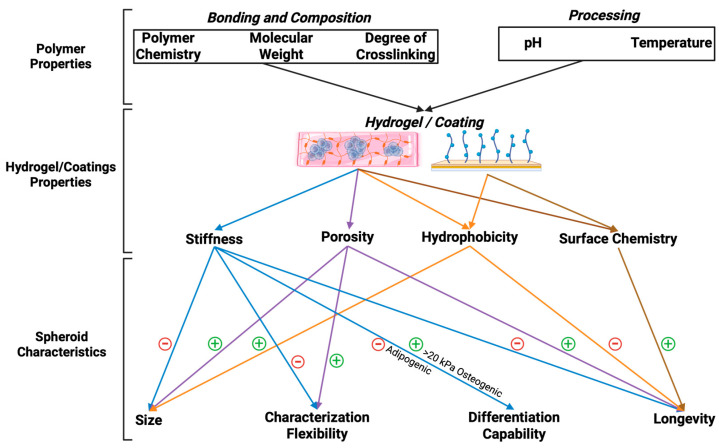
Schematic representation of biomaterial properties, processing parameters, and cellular responses in tissue engineering. Material characteristics (stiffness, porosity, hydrophobicity, surface chemistry) and processing factors (polymer structure, polymerization, temperature) influence spheroid morphology, differentiation, and longevity, ultimately shaping cell–material interactions and tissue regeneration outcomes.

The interplay between biomaterials and cellular microenvironments is a critical determinant in tissue engineering, regenerative medicine, and biomedical applications. Figure 4 shows the multifaceted relationship between biomaterial properties, cellular responses, and processing parameters. These parameters collectively govern the functionality and success of engineered tissue constructs. Fundamental biomaterial characteristics, including stiffness, porosity, hydrophobicity, and surface chemistry, serve as key regulators of cellular behaviors such as adhesion, proliferation, migration, and differentiation. These properties, in turn, directly influence spheroid morphology, differentiation potential, and longevity, which are crucial for maintaining cellular viability and function in three-dimensional culture systems. Additionally, processing parameters, such as polymer molecular structure, the degree of polymerization, and thermal processing, significantly impact the physicochemical properties of biomaterials, further modulating their bioactivity. For instance, the tunability of hydrogel stiffness and porosity can facilitate precise control over nutrient diffusion and cellular infiltration, thereby optimizing the microenvironment for cell growth and matrix deposition. Furthermore, surface modifications, such as functionalization with bioactive ligands, enable enhanced biomimicry of the native ECM, thereby improving cell–material interactions and promoting tissue-specific differentiation.

A comprehensive understanding of the dynamic interactions between biomaterial design and cellular responses is imperative for the rational development of next-generation biomaterials. By leveraging advances in biomaterial science, researchers can engineer constructs that exhibit enhanced biocompatibility, improved mechanical integrity, and optimized biofunctionality for applications in wound healing, organ regeneration, and in vitro disease modeling. Future research should focus on the integration of bioinspired design principles, advanced fabrication techniques, and real-time in vitro and in vivo assessments to further refine biomaterial performance and therapeutic efficacy.

In conclusion, this review article intends to highlight various polymers that are widely used to culture 3D spheroids and the methodology to generate multicellular spheroids. With advancements in MCS culture, the in vitro model can now mimic in vivo properties in many ways. The MCS complex structure aids in the comprehension of cell–cell and cell–matrix interactions. Furthermore, the recapitulation of the in vivo microenvironment by MCSs allows for fundamental research on cancer biology and tissue development, as well as the ability to culture functional tissues in vitro. However, one of the shortcomings of all of the culture techniques and polymers described above is that they have not been explored to study long-term culture and many procedures prepare spheroids that may be lost during media changes. As a result, these approaches may not allow for complete media changes and/or cannot culture adipocytes over an extended period (more than 3 weeks). As a result, in current existing 3D models, cells, for example, adipocytes, fail to achieve a physiologically appropriate outcome of high triglyceride accumulation (i.e., maximal adiposity). Therefore, further research is needed in the development of polymeric coatings that supports the better attachment of the cells to the substrate, thereby allowing the long-term culture of MCSs.

## 6. Future Outlook

While 3D spheroid models have shown significant promise in recapitulating in vivo-like cellular behavior, achieving stable and functional long-term cultures remains a major challenge. Many current systems are unable to maintain spheroid integrity, viability, or phenotypic function beyond two to three weeks. This limitation is particularly evident in adipogenic models, where sustained lipid accumulation and metabolic activity are essential yet difficult to preserve. Future research must therefore focus on the design of polymer systems that support extended culture durations without compromising structural or functional outcomes. This includes developing coatings and scaffolds that provide both initial support for spheroid formation and continued biochemical and mechanical cues that sustain differentiation.

Recent advances in microgel and granular scaffold systems have introduced new opportunities to address these limitations. For example, Caprio et al. demonstrated the integration of injectable mesenchymal stem cell (MSC) spheroids with microgel-based granular composites, which enabled robust cell retention and enhanced structural fidelity during in vivo-like applications [27]. Such composite systems combine the biological advantages of spheroids with the injectability and spatial adaptability of granular scaffolds, supporting scalable, minimally invasive delivery and improved integration with host tissue.

Strategies such as dynamic crosslinking, slow-degrading polymer backbones, and tethered culture approaches may offer avenues to minimize spheroid loss during media changes and maintain cellular phenotype over time. There is also a need for adaptive biomaterials capable of responding to cell-secreted factors, adjusting local stiffness or degradation rates to accommodate tissue maturation. Incorporating matrix remodeling enzymes, oxygen-permeable platforms, or perfusable scaffolds may further enhance long-term nutrient delivery and waste removal, key factors in preventing necrosis or functional decline. Importantly, these innovations should be integrated with real-time imaging, microfluidic platforms, and automated handling systems to enable longitudinal monitoring and scalable screening. Such systems will not only improve experimental reproducibility but also expand the utility of MCSs in modeling chronic diseases, studying developmental biology, and evaluating long-term drug effects.

Looking ahead, the development of synthetic interactive materials represents a paradigm shift in spheroid engineering. These systems go beyond passive scaffolds and are designed to dynamically interface with cells, mimicking certain behaviors of living cells. For instance, artificial cells or protocells, often constructed from functionalized vesicles, can sense environmental cues and release bioactive molecules in response, thus programming cell behavior in real time. Similarly, smart hydrogels that respond to pH, temperature, or matrix metalloproteinases (MMPs) can adapt their stiffness or porosity to accommodate spheroid expansion or differentiation. Bio-orthogonal polymeric coatings, functionalized with ligands or synthetic gene switches, offer further control by selectively activating signaling pathways only under specific biological conditions. Sümbelli et al. recently outlined the potential of such artificial cell-mediated platforms in tissue engineering, emphasizing their ability to orchestrate complex multicellular dynamics and promote spatially regulated tissue formation [97]. Incorporating these synthetic, responsive elements into spheroid systems could enable real-time feedback, adaptive remodeling, and on-demand biochemical signaling, greatly enhancing the physiological relevance and therapeutic potential of MCS-based models. An emerging framework, Spheroid-Hydrogel-Integrated Biomimetic System (SHIBS), represents a systems-level approach that couples the structural fidelity of spheroids with the modular adaptability of synthetic hydrogels. As discussed by Yoo and Lee, SHIBS platforms promise enhanced reproducibility, scalability, and integration with advanced technologies such as microfluidics and AI, setting the stage for next-generation organotypic models [98].

By addressing the fundamental limitations of long-term culture stability and function, the next generation of biomaterial platforms can transform MCSs from short-term models into robust, predictive tools for both research and therapeutic development.

## Figures and Tables

**Table 1:** Characteristics of selected studies for 3D culture of human adipose-derived stem cells using hydrogels.

Polymer	Chemical Structure	Molecular Weight	Processing Condition	Culture Parameters	Culture Period	Purpose
Hyaluronic acid [47]	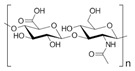	1000 kDa	3%, 4%, 5% *w*/*v* of HA gels with cells (2 × 10^6^, 4 × 10^6^, 6 × 10^6^) in DMEM.	Gels were formed in syringe.	12 h	Stemness study
Hyaluronic acid [48]	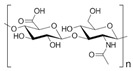	1000 kDa	3% *w*/*v* of HA gel with cells in DMEM; 1–6 × 10^6^ ASCs or 4 × 10^5^ ASCs—4 × 10^4^ ECFCs in 35 mm dish.	Suspended cells were incubated in the presence of HA gel for 24–72 h to form 3D spheroids.	72 h	Angiogenic study
Chitosan with hyaluronic acid [53]	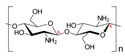	Chitosan: 510 kDa Hyaluronic acid: 2500 kDa	Chitosan membranes were solvent casted using 1% chitosan solution in acetic acid and coated with 0.1, 0.5, or 2.5 mg of HA per cm^2^; hADAS and hPDMC (3 × 10^4^ cells) were seeded on each membrane.	For chitosan membranes, spheroids formed within ~41 h. For chitosan-HA membranes, spheroids formed within ~13 h.	10 days	Stemness study
Chitosan with cartilage ECM [54]	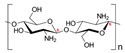		1% chitosan and 0.2% cartilage ECM were mixed and lyophilized to create a porous composite construct and further crosslinked in 0.05% genipin solution; 500,000 ASCs per construct.	Two days after seeding ASCs on the composite scaffold, multiple small ASC spheroids formed on the surface of the composite scaffold.	28 days	Chondrogenic study
PNIPAM hydrogel substrate functionalized with PEG hydrogel [72]	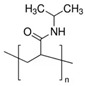	PEG-DA; 575 Da	PNIPAM hydrogel was prepared using free radical polymerization. Photoinduced graft polymerization of PEG-DA was utilized to modify the hydrogel surface.	2.0 × 10^5^ ASCs/cm^2^ were seeded onto the microwell-patterned hydrogel.	5 days	Spheroid formation

**Table 2:** Characteristics of selected studies for 3D culture of human adipose-derived stem cells using ultra-low/low adhesion or concave multi-well.

Polymer	Chemical Structure	Processing Condition	Culture Parameters	Culture Time	Purpose
Agarose [80]	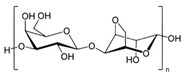	PDMS stamps were placed in 3% agarose solution and an agarose well of 400 μm diameter and 200 μm deep was created; 1.5 million cells per agarose chip; centrifuged briefly at 1500 rpm.	The cells formed spheroids in 24 h.	7 days	Study of osteogenic differentiation
Agarose [81,82]	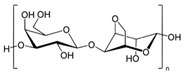	2% agarose powder mixed with LG-DMEM medium was pipetted into 400 μm diameter rubber micro-molds; 3.5 × 10^5^ hASCs per micro-well.	After 3 days, almost all hASCs were generated into spheroids.	Spheroids were transferred to new plastic Petri dishes for 5 days of culture before 2 weeks of differentiation	Study of adipogenic, osteogenic, and neurogenic differentiation
Agarose [83]	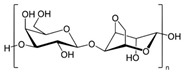	2% sterile agarose solution was pipetted onto the master micro-molds, and the newly formed agarose molds transferred into a polystyrene Petri dish. Agarose hydrogel micro-mold with 35 concave wells of 800 μm diameter and 800 μm depth.	To achieve spheroids with radii of 115, 135, 175, and 215 μm, cell solutions of 0.5, 1.0, 2.5, and 5.0 million hASCs/mL were used.	3 days	Cell ischemic model
Agarose [84]	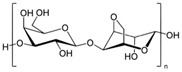	3% *w*/*v* non-adhesive agarose microwell consisting of 2865 pores with 200 μm diameter and depth of 220 μm.	7.5 × 10^5^ cells were seeded onto the microwell, resulting in approximately 262 cells per pore. For angiogenic study, 1.0 × 10^6^ cells (350 cells per pore) were used.	10 days	Angiogenic study in monoculture and co-culture ASC/HUVEC/HFF
PDMS coated with BSA [85]	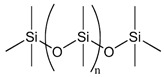	3% *w*/*v* BSA-coated PDMS concave microwell of 300 mm diameter containing 64 holes.	1 × 10^5^ ASCs per well were seeded and incubated at 37 °C for 24 h.	1 day	Regeneration of lung tissue
PDMS coated with BSA [86]	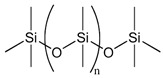	3% *w*/*v* BSA-coated PDMS concave microwell of 300 mm.	Human hepatocyte spheroids. Co-cultured spheroids with 1:1 human hepatocytes and hASCs.	7 days	To study the effects of co-culturing of hepatocytes with adipose-derived stem cells on hepatocyte function and spheroid formation
Pluronic F127 coated well plate [90]	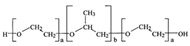	10% *w*/*v* aqueous solution of Pluronic F127 was coated on a 96-well plate.	200 µL, 5 × 10^4^ ASCs/mL per well. Plate was placed on a rotary shaker with gentle rotation. The 3D spheroids formed after approximately 24–48 h from seeding.	7 days	Osteogenic study

## Abbreviations

3D	Three-dimensional
2D	Two-dimensional
MCSs	Multicellular spheroids
ECM	Extracellular matrix
RGD	Arginine–glycine–aspartic acid
ULA	Ultra-low attachment
PDMS	Polydimethylsiloxane
PEG	Polyethylene glycol
MMPs	Matrix metalloproteinases
VEGF	Vascular endothelial growth factor
TGF-β	Transforming growth factor-β
GFOGER	Glycine–phenylalanine–hydroxyproline–glycine–glutamic acid–arginine
ELP	Elastin-like polypeptide
hASCs	Human adipose-derived stem cells
HA	Hyaluronic acid
GBM	Glioblastoma
GSCs	Glioblastoma stem cells
PEGDA	PEG-diacrylate
PF	PEGDA-fibrinogen
PLGA	Poly(lactic-co-glycolic acid)
IL-8	Interleukin-8
PNIPAM	Poly(N-isopropylacrylamide)
AA	Acrylic acid
hPSCs	Human pluripotent stem cells
LG-DMEM	Low-glucose Dulbecco’s Modified Eagle’s Medium
HUVECs	Human Umbilical Vein Endothelial Cells
HFFs	Human foreskin fibroblasts
BSA	Bovine serum albumin
PF127	Pluronic F127
PEO	Poly(ethylene oxide)
PPO	Poly(propylene oxide)
CVD	Chemical vapor deposition
NHS	N-hydroxysuccinimide
FGF-2	Fibroblast growth factor-2
TCPS	Tissue culture polystyrene
pV4D4	Poly(2,4,6,8-tetravinyl-2,4,6,8-tetramethyl cyclotetrasiloxane)
iCVD	Initiated chemical vapor deposition
CSCs	Cancer stem cells
hMSCs	Human mesenchymal stem cells
PEI	Polyethyleneimine
OA	Oleic acid
LA	Linoleic acid
